# Gestational Diabetes Mellitus Across the Perinatal Continuum: A Narrative Review of Woman-Centered, Holistic Care Models

**DOI:** 10.3390/healthcare14121791

**Published:** 2026-06-21

**Authors:** Eleftheria Lazarou, Dimitra Metallinou, Ourania Kolokotroni, Ekaterini Lambrinou, Panagiota Miltiadous, Georgios Papaetis, Andri Evripidou, Konstantinos Mikellidis, Charilaos Kontos, Spyridakis Chrysostomou, Michalis Chrysostomou, Charalambos Neocleous, Elli Parpa, Constantina Constantinou, Eleni Hadjigeorgiou

**Affiliations:** 1 Department of Obstetrics, Iasis Private Hospital, Paphos 8069, Cyprus; antri.evripidou@cytanet.com.cy (A.E.); dr.c.kontos@hotmail.com (C.K.); elli.p2001@gmail.com (E.P.); konstantinakonstantinou78@gmail.com (C.C.); 2 Department of Nursing, School of Health Sciences, Cyprus University of Technology, Limassol 3036, Cyprus; o.kolokotroni@cut.ac.cy (O.K.); ekaterini.lambrinou@cut.ac.cy (E.L.); panagiota.miltiadous@cut.ac.cy (P.M.); eleni.hadjigeorgiou@cut.ac.cy (E.H.); 3 Department of Midwifery, School of Health and Care Sciences, University of West Attica, 12243 Athens, Greece; dmetallinou@uniwa.gr; 4 Internal Medicine and Diabetes Clinic, K.M.P. THERAPIS Paphos Medical Center, Paphos 8010, Cyprus; gpapaetis@yahoo.gr (G.P.); constantinos1926@hotmail.com (K.M.); 5 IASO Medical Centre, Limassol 3026, Cyprus; dakis@iasoivf.com (S.C.); michali1@cytanet.com.cy (M.C.); 6 Department of Nursing, Frederick University of Cyprus, Nicosia 1036, Cyprus; hsc.cn@frederick.ac.cy

**Keywords:** gestational diabetes, GDM, woman-centered care, perinatal continuum, pregnancy, holistic-care, education, screening, well-being

## Abstract

**Highlights:**

**What are the main findings?**

- Gestational Diabetes Mellitus (GDM) management requires a multidisciplinary, woman-centered approach that integrates clinical treatment with antenatal education, empathetic communication, psychological support, and lifestyle interventions.
- Structured antenatal education and family involvement improve women’s understanding of GDM and support adherence to dietary, physical activity, and glucose-monitoring recommendations.

**What are the implications of the main findings?**

- Policies should promote woman-centered, holistic GDM care, including training healthcare professionals in empathetic communication and implementing structured, multidisciplinary care pathways that integrate preventative, educational, and supportive interventions.
- Strengthening postpartum follow-up, lifestyle counseling, family education, and implementing reminder systems can help reduce the risk of progression to type 2 diabetes in women with a history of GDM.

**Abstract:**

Gestational Diabetes Mellitus (GDM) represents a significant public health concern due to its association with adverse maternal and neonatal outcomes, as well as elevated long-term metabolic risks. Its prevalence varies substantially depending on the diagnostic criteria used and the population studied. Women with GDM frequently experience heightened stress, anxiety, and uncertainty, underscoring the need for accessible information, counseling, and ongoing support to navigate glucose monitoring, dietary adjustments, and treatment regimens. Although clinical management has been extensively studied, research has largely focused on metabolic monitoring and therapeutic interventions, often underemphasizing prevention strategies, women’s informational needs, and maternal psychological well-being. Emerging evidence and international guidelines increasingly advocate for integrating these components into structured, woman-centered GDM care plans that actively involve families. Such approaches empower women to engage in self-management, enhance health literacy, support adherence to lifestyle and pharmacological interventions, and promote sustainable behavioral changes. This narrative review presents a comprehensive, holistic model of care across the perinatal continuum, emphasizing early risk identification, preventive strategies, and multidisciplinary coordination. Core elements include individualized antenatal education, empathetic communication, and family engagement, fostering self-efficacy, continuity of care, and integration of medical, educational, and psychosocial interventions. Equipping healthcare professionals with the competencies to deliver this holistic, woman-centered framework is essential to optimize maternal and neonatal outcomes and mitigate the long-term health consequences of GDM.

## 1. Introduction

Gestational Diabetes Mellitus (GDM) represents an increasingly important global public health concern, with prevalence rates rising steadily in recent years [1]. According to the International Diabetes Federation (IDF), GDM affects approximately one in six pregnancies worldwide, with current estimates largely based on the diagnostic criteria proposed by the International Association of Diabetes and Pregnancy Study Groups (IADPSG). Nevertheless, reported prevalence rates vary considerably across studies, and accurate estimation of the true burden of GDM remains challenging due to differences in diagnostic criteria, population characteristics, and underlying metabolic risk profiles [2]. Furthermore, disparities in socioeconomic status and healthcare access play a critical role in shaping regional variation in prevalence. In low-income countries, limited access to healthcare services and diagnostic facilities frequently leads to underdiagnosis, thereby underestimating the true burden of disease [3]. Collectively, these factors contribute to substantial heterogeneity in reported prevalence across regions, with estimates ranging from 10.2% in the Middle East and North Africa to 24.9% in South-East Asia, and intermediate values reported in Europe (13.9%), Africa (10.8%), the Western Pacific (17.5%), and North America and the Caribbean (17.9%) [2].

The condition is widely recognized as a high-risk pregnancy complication due to its well-established association with adverse short- and long-term maternal and neonatal outcomes [4]. Therefore, the maternal metabolic environment during pregnancy plays a crucial role in shaping the long-term health trajectory of the offspring, as it may permanently influence physiological and metabolic pathways [5,6]. Consequently, children born to mothers with poorly controlled GDM are at greater risk of developing metabolic complications later in life. Similarly, a history of GDM is consistently identified as one of the strongest predictors of subsequent Type 2 Diabetes Mellitus (T2DM) in mothers, highlighting the importance of preventive strategies and long-term screening [7]. Beyond its biological consequences GDM can substantially impact women’s psychosocial well-being during the perinatal period [8,9]. The communication of a GDM diagnosis requires sensitivity and a woman-centered approach, as the way in which the diagnosis is delivered can significantly influence a woman’s psychological adjustment and engagement with management strategies [10].

Given its increasing prevalence and long-term health implications, GDM has become a significant public health priority, making prevention and management central to contemporary healthcare practice [1]. Despite extensive research on clinical management strategies, a notable gap remains in comprehensive reviews examining the full perinatal continuum of care. In particular, limited attention has been given to structured antenatal education, psychological support, family involvement in care processes, and the promotion of maternal well-being [11,12].

This review therefore aims to address these gaps by highlighting dimensions of care that extend beyond clinical management while placing the woman at the center of the care process. By emphasizing preventative, educational, psychological, and supportive interventions, the proposed framework integrates specialized medical management of GDM within a holistic approach that promotes prevention strategies, maternal physiological health, and overall well-being. Given that this review incorporates care pathways and clinical algorithms, it is important to clarify that these are intended as an integrative synthesis of existing evidence and guideline recommendations, reflecting a comprehensive and woman-centered approach to care, rather than formally developed clinical guidelines produced through a standardized guideline development methodology.

## 2. Methods

### 2.1. Study Design

This study employed a narrative review design to synthesize current evidence on the holistic management of GDM across the perinatal continuum, with particular emphasis on woman-centered care and clinically applicable strategies. A narrative approach was selected to enable the integration of diverse forms of evidence, including primary research studies, contemporary clinical guidelines, and health system frameworks, which may not be fully captured through a strictly systematic methodology. The methodological conduct and reporting of this narrative review were informed by the SANRA (Scale for the Assessment of Narrative Review Articles) framework to enhance transparency, methodological rigor, and consistency in the synthesis and interpretation of the evidence [13].

### 2.2. Search Strategy

A comprehensive literature search (2015–2026) was conducted using electronic databases, including PubMed/MEDLINE, Scopus, and CINAHL, supplemented by targeted screening of guideline repositories from the IDF, American Diabetes Association (ADA), American College of Obstetricians and Gynecologists (ACOG), National Institute for Health and Care Excellence (NICE), and the World Health Organization (WHO), as well as other relevant professional organizations, in order to synthesize the most recent clinical guidelines.

The search strategy combined Medical Subject Headings (MeSH) and free-text keywords related to GDM and its continuum of care. Representative search terms included “gestational diabetes mellitus,” “prevention,” “preconception care,” “screening,” “diagnosis,” “management,” “neonatal care,” “multidisciplinary team,” “antenatal care,” “intrapartum care,” “postpartum follow-up,” “holistic care,” “woman-centered care,” “guidelines,” “breastfeeding,” and “long-term outcomes.”

These terms were combined using Boolean operators (“AND,” “OR,” “NOT”), and search strategies were adapted to the specifications of each database. Additional targeted searches were conducted to identify key clinical guidelines, consensus statements, and emerging evidence. Furthermore, reference lists of included studies were manually screened to ensure comprehensive coverage of the relevant literature. The literature search was completed on 10 February 2026.

### 2.3. Study Selection and Data Synthesis

Study selection was conducted through screening of titles, abstracts, and full-text articles for relevance to the review objectives and recency of evidence. Given the narrative design, no rigid exclusion criteria were applied; however, priority was given to contemporary clinical guidelines, high-quality studies, and clinically relevant evidence. Included studies and guidelines were selected based on their relevance to prevention, prediction, diagnosis, holistic management strategies, and postpartum follow-up. Emerging evidence from innovative, evolving, and under-researched areas was also included where relevant, to provide a comprehensive synthesis and inform the scientific and clinical community, while acknowledging that these areas are not yet fully established or clinically validated.

Data synthesis followed a thematic approach, enabling the integration and interpretation of evidence from diverse sources. The included literature was systematically organized and analyzed across key stages of the perinatal continuum. Findings were compared and critically interpreted to identify areas of convergence, divergence, and gaps in the current evidence base, with consideration of clinical and contextual applicability.

A multidisciplinary approach was adopted during the synthesis process, with contributing authors providing domain-specific expertise across the principal areas of care addressed in the review. Title and abstract screening, as well as full-text assessment, were conducted independently by two reviewers. Any discrepancies or uncertainties arising during the selection and evaluation process were resolved through discussion and consensus with a third reviewer, appointed according to the relevant thematic section and corresponding clinical expertise. This approach strengthened the clinical applicability and interpretative rigor of the review while ensuring methodological consistency across sections.

The findings were subsequently structured into four overarching thematic domains: (1) prevention strategies, (2) antepartum care and management, (3) intrapartum care and management, and (4) postpartum follow-up and long-term care.

## 3. Prevention Strategies

### 3.1. Risk Profiling

**Figure 1:**
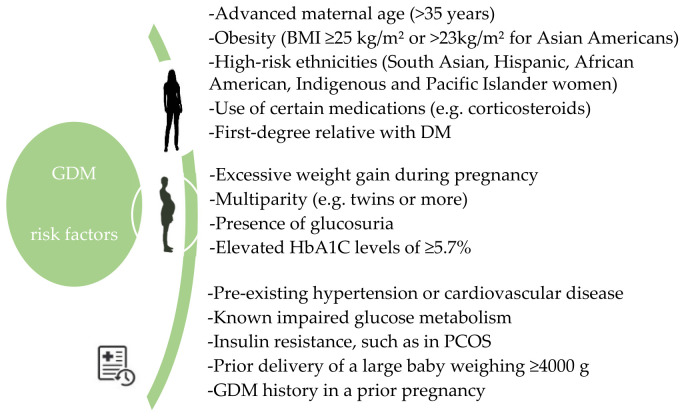
GDM risk factors and high-risk populations [18,25,26,27]. Abbreviations: BMI (Body Mass Index); DM (Diabetes Mellitus); GDM (Gestational Diabetes Mellitus); HbA1c (Hemoglobin A1c); PCOS (Polycystic Ovary Syndrome).

The pathophysiology of GDM is a complex metabolic phenomenon, arising from a failure of maternal adaptive mechanisms to meet the heightened insulin demands of pregnancy [14]. Although the precise molecular pathways are still being elucidated, GDM is widely recognized to develop when a woman’s pancreatic beta-cell compensatory capacity is insufficient to overcome the physiological insulin resistance inherent to gestation [14,15]. Beyond these inherent physiological adaptations, multiple modifiable and non-modifiable risk factors further increase the likelihood of developing GDM (Figure 1) [16,17].

Risk assessment is a fundamental component of early prenatal care and plays a crucial role in the prevention and early detection of GDM [18]. Accordingly, during the initial prenatal consultation, health care professionals (HCPs) should conduct a systematic evaluation of both modifiable and non-modifiable risk factors [1,19]. Specifically, maternal age is a key predictor, with women over 35 years experiencing a progressively higher risk [17,20]. Pre-pregnancy overweight (Body Mass Index [BMI] ≥ 25 kg/m^2^) or obesity (BMI ≥ 30 kg/m^2^) significantly increases risk due to baseline insulin resistance [21,22], while the risk is further amplified by excessive gestational weight gain and a sedentary lifestyle. Additionally, a woman’s clinical history is a vital indicator; those with pre-existing impaired glucose tolerance or a prior history of GDM carry a markedly increased likelihood of recurrence in subsequent pregnancies [22]. Early identification of these factors enables the implementation of targeted preventive strategies, including individualized nutritional counseling, guidance on appropriate gestational weight gain, and the promotion of regular physical activity [23,24]. A proactive risk assessment approach also provides an opportunity to deliver antenatal education and promote behavioral modifications that may improve both maternal and neonatal outcomes [24].

The potential for early intervention is increasingly supported by emerging evidence, which suggests that between 27% and 66% of GDM cases are identifiable during early pregnancy, depending on the population and criteria applied [1]. Capitalizing on this early diagnostic window requires accessible healthcare pathways; indeed, recent evidence highlights that midwifery and community-based care initiatives are instrumental in facilitating these early risk assessments, thereby ensuring timely diagnosis and proactive management [25,26].

### 3.2. Preconception Care

High-risk populations should undergo preconception consultations and diabetes mellitus (DM) screening and receive appropriate care to detect and manage hyperglycemia prior to conception, as early identification and treatment of elevated glucose levels may reduce the risk of congenital anomalies and other adverse maternal and fetal outcomes [18]. HCPs should address social determinants that contribute to metabolic disparities [28] and provide preconception lifestyle modifications and targeted education, which are essential for lowering the incidence of GDM [24,29]. Routine care should include counseling on nutrition, physical activity, and healthy weight management (Table 1), as pre-pregnancy BMI is a major determinant of GDM risk [30]. Women with a BMI ≥ 25 kg/m^2^ derive the greatest benefit from early interventions, including adopting a nutrient-dense diet and increasing physical activity, which can stabilize plasma glucose levels and mitigate excessive gestational weight gain [31].

According to the updated ADA (2026) recommendations [18], preconception care for individuals with prediabetes, pre-existing DM, or a history of GDM should emphasize nutritional assessment and counseling, lifestyle guidance, meal planning, correction of deficiencies, safe food practices, and structured DM self-management education, including preconception glycemic targets, progression of insulin resistance, and potential pregnancy risks. Family planning, including the use of long-acting reversible contraception until pregnancy is desired, should also be discussed. Preconception care extends beyond individual counseling; it also strengthens public health by empowering women to make informed decisions that safeguard their long-term metabolic health and that of their offspring, including the prevention of GDM [18,24].

### 3.3. Emerging Preventive Strategies

Emerging evidence suggests that inositol supplementation may represent a promising preventive strategy for GDM. Inositol is thought to improve insulin sensitivity and glucose metabolism and has been shown to reduce hyperinsulinemia and improve ovarian function in women with polycystic ovary syndrome (PCOS), a condition strongly associated with an increased risk of developing GDM [33]. Given the important role of insulin resistance in the pathophysiology of GDM, these findings have generated growing interest in the potential role of inositol during pregnancy.

Several studies have reported that inositol supplementation during early pregnancy may reduce both the incidence and severity of GDM, particularly among women considered to be at increased metabolic risk. In addition to its potential metabolic benefits, inositol has generally demonstrated a favorable safety profile and good tolerability, making it an attractive candidate for preventive intervention [33,34]. Despite these encouraging findings, the available evidence remains heterogeneous, with variations in study populations, supplementation regimens, and outcome measures across studies. Consequently, further large-scale, high-quality randomized controlled trials are required to better establish its efficacy, determine optimal dosing strategies, and evaluate long-term maternal and neonatal outcomes before routine clinical implementation can be recommended [34].

## 4. Antepartum Care and Management

Management of GDM during pregnancy requires a holistic approach that addresses not only blood glucose control but also the emotional, nutritional, and social needs of the pregnant woman [8,9,35,36]. This approach integrates medical therapy, personalized nutrition and physical activity guidance, psychosocial support, and woman education to promote maternal and fetal well-being [21,24]. By considering the physical, psychological, and lifestyle aspects of care, holistic management supports adherence, reduces complications, and empowers women to participate actively in their health throughout pregnancy [24,36]. Effective GDM management requires a synchronized, tiered approach where the midwife, endocrinologist, obstetrician, neonatologist and nutritionist maintain a constant, parallel presence throughout the perinatal continuum [12,28,37]. Since 70% to 85% of women achieve metabolic control through lifestyle modifications alone [37], the midwife acts as the primary lead for these non-pharmacological strategies. In this capacity, the midwife serves as the vital link who prevents unnecessary medicalization, providing essential education and psychological support to mitigate the emotional burden and fear often associated with the diagnosis [12]. The endocrinologist and obstetrician provide essential, ongoing surveillance for all gestations, offering the specialized clinical authority required for the 15% to 30% of women who eventually necessitate pharmacological titration [37,38]. Simultaneously, nutritionists deliver the medical nutrition therapy fundamental to achieving glycemic stability [28,39], while the neonatologist ensures specialized neonatal surveillance and immediate care at the time of labor to manage potential metabolic complications in the newborn [40].

By bridging the gap between high-level clinical surveillance and the woman’s daily lived experience, this multidisciplinary framework respects the specialized expertise of each profession, ensuring that clinical targets are met through maternal empowerment and informed self-management.

### 4.1. Screening and Diagnosis of GDM

GDM is diagnosed when Oral Glucose Tolerance Test (OGTT) results exceed one or more established glucose thresholds [18]. However, screening recommendations and diagnostic criteria vary considerably across major international guidelines, including those proposed by the IADPSG (2010) [41], NICE (2015) [42], and Australasian Diabetes in Pregnancy Society (ADIPS) (2025) [43]. These differences include variations in screening strategies, diagnostic threshold values, and the number of abnormal glucose measurements required to establish the diagnosis [44]. Furthermore, screening practices differ internationally, with some regions adopting selective risk-based screening approaches, while others recommend universal screening [42,43].

More specifically, NICE (2015) [42] recommends a selective screening strategy in high-risk women, using a 75 g OGTT performed between 24 and 28 weeks of gestational age (GA). Diagnosis is established when fasting plasma glucose is ≥5.6 mmol/L and/or 2 h plasma glucose is ≥7.8 mmol/L. In contrast, the IADPSG recommends universal screening using a 75 g OGTT with fasting, 1 h, and 2 h measurements, applying diagnostic thresholds of 92 mg/dL, 180 mg/dL, and 153 mg/dL, respectively [41]. Despite these differences, OGTT screening is generally recommended between 24 and 28 weeks of GA [18,41,45]. Nevertheless, the screening process should begin with a comprehensive risk assessment during the initial prenatal visit in order to identify high-risk individuals [18,43]. In women considered to be at high risk, early screening may be indicated; however, approaches to early screening vary considerably across regions and guideline frameworks. Specifically, some organizations support early OGTT screening in high-risk women followed by repeat testing later in pregnancy, whereas others do not recommend routine early screening for GDM [41,42,43].

The ADA recommends early evaluation of high-risk individuals either before conception or prior to 15 weeks of GA in order to identify previously undiagnosed DM or abnormal glucose metabolism [18]. Routine screening for GDM is subsequently recommended between 24 and 28 weeks of GA in women without evidence of overt DM earlier in pregnancy. Similarly, the IADPSG supports early assessment for overt DM in pregnancy [41]. Conversely, ADIPS (2025) [43] recommends early OGTT screening before 20 weeks of GA, ideally between 10 and 14 weeks, in women with a previous history of GDM, HbA1c levels between 6.0% and 6.4%, or when individualized risk assessment indicates increased risk.

**Figure 2:**
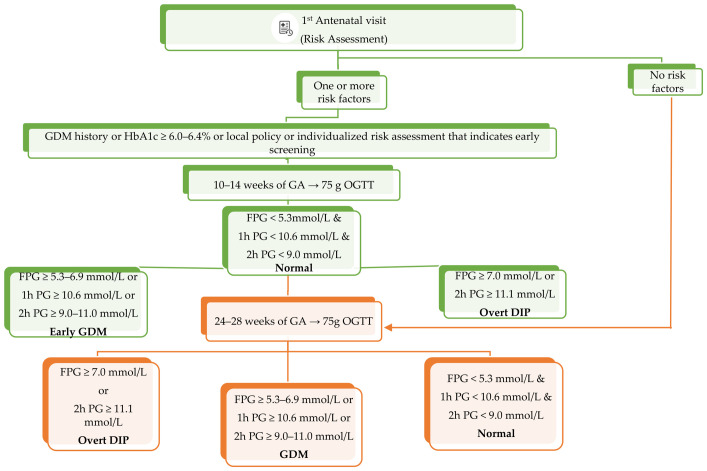
GDM diagnostic and management algorithm based on the ADIPS (2025) recommendations [43]. *Explanation:* Risk assessment should be performed at the first antenatal visit. In the absence of risk factors, a 75 g OGTT is recommended at 24–28 weeks of GA; however, in women with previous GDM, HbA1c ≥ 6.0–6.4%, or when early screening is indicated based on local policy or individualized risk assessment, a 75 g OGTT should be performed before 20 weeks of GA (ideally at 10–14 weeks). If the result is normal, the OGTT should be repeated at 24–28 weeks of GA. In the absence of risk factors, a diagnostic 75 g OGTT is standardly recommended at 24–28 weeks of GA. Abbreviations: 1 h (one-hour); 2 h (two-hour); DIP (Diabetes in Pregnancy; FPG (Fasting Plasma Glucose); GA (Gestational Age); GDM (Gestational Diabetes Mellitus); HbA1c (Hemoglobin A1c); OGTT (Oral Glucose Tolerance Test); PG (Plasma Glucose).

Although the OGTT is widely regarded as the diagnostic gold standard, screening pathways and diagnostic thresholds differ substantially between guidelines. Consequently, Figure 2 reflects one contemporary evidence-based approach [43], rather than a universally adopted standard. Implementation of these pathways may therefore require adaptation according to local healthcare systems and regional screening policies.

Given that OGTT testing may be burdensome for some women, decisions regarding repeat testing should be individualized and guided by a woman-centered, culturally safe, and holistic model of care. Active participation of women in shared decision-making should be encouraged and supported by evidence-based, culturally appropriate, and linguistically accessible educational resources that clearly communicate the rationale, benefits, and implications of GDM screening and diagnosis [24,43].

### 4.2. Emerging Diagnostic and Predictive Biomarkers for GDM

Biomarkers are measurable biochemical indicators detectable in biological fluids such as serum, urine, and saliva [46]. In clinical medicine, they are essential tools for disease detection and risk prediction, enhancing understanding of underlying pathophysiological mechanisms, monitoring therapeutic response, and estimating future health outcomes [15]. In light of the OGTT limitations [44], there has been growing scientific interest in alternative approaches for the early identification of GDM [47,48].

The discovery of reliable early pregnancy biomarkers could significantly improve antenatal care by enabling early risk stratification and facilitating targeted interventions before the onset of maternal hyperglycemia and related complications [49,50]. Early identification of at-risk women would allow for timely implementation of lifestyle modification, closer clinical surveillance, and, where appropriate, pharmacological intervention to delay or prevent disease progression [41,50].

In recent years, extensive research has explored a broad range of biomarkers associated with GDM, including inflammatory and hematological biomarkers, indicators of insulin resistance, lipid metabolism profiles, and sex hormones [49,51]. Some biomarkers have been investigated primarily in early pregnancy for their ability to predict subsequent development of GDM [52,53], whereas others appear to be more relevant later in gestation and may serve as potential adjuncts or alternatives to the OGTT [54,55].

Within this expanding field, a range of candidate biomarkers has been proposed—including adiponectin, pregnancy-associated plasma protein A (PAPP-A) [49,53], glycated albumin [52], betatrophin [54], high mobility group box 1 (HMGB1) [55], and various lipid-related indices [51]. Collectively, these markers reflect distinct but interrelated pathophysiological pathways. Investigations in early pregnancy have largely focused on first-trimester concentrations of adiponectin and PAPP-A; notably, Wang et al. (2024) demonstrated significantly lower levels of both markers in women who later developed GDM compared with normoglycemic controls [53]. However, the data for PAPP-A are conflicting, as a recent meta-analysis by Li et al. (2026) revealed only a non-significant trend toward lower levels alongside marked inter-study heterogeneity [49]. Moving into mid-gestation, glycated albumin emerges as another potential indicator, though counterintuitively, evidence suggests it may be reduced prior to clinical diagnosis. Specifically, in a cohort of 321 participants, Cole et al. (2024) reported lower plasma glycated albumin concentrations during 15–26 weeks of GA in GDM, though the underlying mechanisms driving this reduction remain unclear [52].

Emerging evidence also implicates inflammatory mediators such as HMGB1, with Giacobbe et al. (2016) reporting significantly elevated serum concentrations in women with GDM during late pregnancy [55]. Remarkably, this difference persisted even after adjusting for maternal age and pre-pregnancy BMI. While these findings point to HMGB1 as a key player in the inflammatory milieu of GDM, larger-scale prospective studies are warranted to validate these results and determine its predictive value in early gestation.

In parallel, metabolic profiling studies have highlighted the relevance of lipid biomarkers in early pregnancy. According to the Swinburne et al. (2025) data synthesis, total cholesterol, triglycerides, low-density lipoprotein cholesterol, and high-density lipoprotein cholesterol have all been examined in relation to GDM risk, with elevated triglyceride concentrations emerging as the most consistent finding among women who later develop the condition [51].

Recognizing the multifactorial nature of GDM, several studies have also developed predictive models that combine maternal characteristics and pregnancy-related risk factors with panels of biomarkers to improve early risk stratification and detection [56]. Early identification of women at high risk remains essential for optimizing maternal and fetal outcomes [57]. Although the emerging evidence is promising, findings across studies remain inconsistent, and no single biomarker has yet demonstrated sufficient diagnostic accuracy or clinical utility for routine implementation in practice.

### 4.3. Communication of GDM Diagnosis

The manner in which a diagnosis of GDM is communicated plays a critical role in shaping a woman’s understanding, emotional response, and engagement with management. Research indicates that many women experience anxiety and confusion immediately after being diagnosed, particularly when information is delivered in a rushed or overly technical manner [58]. A recent qualitative study highlighted that women value clear, empathetic explanations about the condition and its implications for maternal and fetal health. The study also emphasized the importance of supportive communication and practical guidance for self-management at the time of diagnosis. Evidence suggests that effective communication during the diagnostic encounter improves women’s comprehension, emotional adjustment, and self-efficacy, ultimately facilitating adherence to lifestyle and treatment recommendations. These findings underscore the importance of which HCPs should use simple, non-technical language, explain the implications for both maternal and fetal health, and outline practical steps for management, including lifestyle modifications and, if needed, pharmacologic therapy. It is essential to assess the women’s understanding and emotional response, provide space for questions, and acknowledge concerns about potential risks and treatment burden [10].

### 4.4. Psychological Support and Well-Being

The diagnosis of GDM can be particularly overwhelming, as reported by Carolan-Olah et al. (2017) [59], and early guidance and support from HCPs are essential to address these concerns [60]. Women diagnosed with GDM are at heightened risk of prenatal anxiety, stress, and depression, often due to limited information and difficulties understanding daily management, treatment, and care requirements [9,61,62]. Contributing factors include blood glucose self-monitoring, dietary restrictions, and potential initiation of insulin therapy [63]. Women with prior education about GDM or previous experience of the condition are generally better equipped to understand the diagnosis and cope effectively [10].

HCPs should be adequately trained and aware of the psychological and educational needs of women with GDM [9,10]. They should allocate sufficient time to address questions, clarify concerns, and provide comprehensive explanations, or refer women to appropriate specialists or support services when necessary [59]. Such approaches foster trust in the healthcare team, enhance adherence to management and preventive strategies, and ensure consistent guidance. Support through counseling, structured education, cognitive-behavioral strategies, and peer support can help women manage stress, improve treatment adherence, and maintain overall well-being [10,24]. By addressing both the emotional and physical aspects of GDM, HCPs can provide comprehensive care that supports healthier pregnancies and better outcomes for mothers and offspring [35,59].

### 4.5. Family Involvement in GDM Care

Family plays an important role in the management of GDM, as many of the recommended lifestyle modifications and self-management behaviors occur within the home environment [10]. More specifically, following diagnosis, women are often required to implement substantial behavioral changes, including dietary modifications, regular physical activity, frequent glucose monitoring, and sometimes pharmacological therapy. These changes may be difficult to sustain without a supportive social context, highlighting the importance of partner and family engagement in the management process [10,35].

Research indicates that women with adequate family support demonstrate improved adherence to dietary recommendations, glucose monitoring routines, and physical activity guidelines compared with those who lack such support [64]. Partners and family members can assist in practical ways, such as supporting healthy meal preparation, encouraging physical activity, and helping women navigate the emotional challenges associated with a high-risk pregnancy. Conversely, limited understanding of GDM among family members may create barriers to effective self-management, particularly when traditional household dietary patterns conflict with recommended nutritional strategies [10,60].

In addition, involving family members in antenatal education programs has been shown to improve understanding of the condition and facilitate supportive behavioral changes within the household. Educational interventions that include partners provide an opportunity for HCPs to explain the pathophysiology of GDM, its potential maternal and fetal consequences, and the importance of adherence to recommended management strategies [24,65]. Such family-centered education can strengthen the home support system and contribute to improved glycemic control and pregnancy outcomes.

Beyond practical assistance, family involvement also provides emotional support that can help mitigate the psychological burden associated with GDM [60]. Women diagnosed with the condition frequently report feelings of anxiety, stress, and guilt related to concerns about fetal health and the demands of daily disease management [9,10]. Emotional encouragement from partners and family members can enhance coping capacity, reduce psychological distress, and increase confidence in self-management practices [58,60].

Family-centered support may also extend into the postpartum period, which is a critical window for long-term metabolic risk prevention. Women with a history of GDM have a substantially increased risk of developing T2DM later in life, making sustained lifestyle modification essential [1]. Educating partners and families about these long-term risks can encourage continued healthy behaviors within the household, thereby supporting postpartum weight management, physical activity, and adherence to recommended glucose screening [10,66].

Consequently, integrating family involvement into GDM care aligns with holistic and woman-centered models of maternity care recommended by organizations [18,24]. Encouraging partner participation in educational sessions, counseling, and postpartum follow-up programs can strengthen support networks and promote sustainable health behaviors that benefit both maternal and neonatal outcomes [65].

### 4.6. Antenatal Education

All pregnant women should receive antenatal education and information during pregnancy and prior to labor, as access to accurate, high-quality information is a cornerstone of GDM management [18,24]. Educational resources are most effective when tailored to women’s needs, using clear, culturally appropriate language that facilitates understanding of the risks, potential complications, and long-term consequences of GDM [10,58,67]. Providing such individualized information empowers women to make informed decisions, adhere to lifestyle and treatment recommendations, and actively participate in the management of their condition [10].

Emerging evidence and guidance from international organizations emphasize the inclusion of structured antenatal education in GDM care plans (Table 2). Education should cover the effects of GDM on pregnancy, diet and physical activity, weight management, and glycemic control as part of comprehensive antenatal care. Effective programs integrate sustainable lifestyle habits with technical skills in glucose monitoring and insulin administration [24,68] and begin with preconception weight management and promotion of appropriate gestational weight gain tailored to pre-pregnancy BMI [24].

Central to this framework is the development of glycemic literacy, where women learn self-management techniques and the clinical significance of maintaining plasma glucose within target ranges. Structured guidance on healthy diet and regular physical activity enhances insulin sensitivity and may reduce the need for pharmacological interventions [18,24,68]. Explaining the complex nature of GDM and its potential effects on maternal, fetal, and neonatal health provides transparency, reduces anxiety, and fosters engagement with the care plan [24].

A unique strength of this approach is the inclusion of the woman’s family in the educational process, creating a supportive domestic environment that facilitates sustained behavioral change and reduces psychological burden [10]. Education is most effective when culturally sensitive, tailored to health literacy, and reinforced throughout the perinatal continuum, ensuring women are empowered to manage their metabolic health and protect long-term outcomes [24]. Programs incorporating tailored guidance on nutrition, physical activity, glucose monitoring, and lifestyle modification have demonstrated significant benefits, including reduced complications such as preterm labor, macrosomia, neonatal hypoglycemia, and respiratory issues compared with standard care [69].

### 4.7. Nutritional Therapy and Physical Activity

All pregnant women diagnosed with GDM should be referred to a nutritionist for individualized dietary planning. This approach is essential, as it must consider preconception weight, multiple gestations, baseline health status, and existing dietary habits [68,70]. To achieve stable glycemic control, a structured dietary regimen is recommended, typically involving 6–8 small, evenly spaced meals per day to prevent postprandial glucose spikes and maintain metabolic equilibrium [68]. Dietary composition should emphasize low-glycemic index carbohydrates (approximately 35–45% of total caloric intake), healthy fats, and 20% protein [71,72]. As pregnancy progresses, nutritional requirements evolve, necessitating continuous titration of the care plan based on self-monitored glucose values, weight gain patterns, physical activity, and concurrent pharmacological treatments.

Complementing nutrition therapy, physical activity is a key first-line intervention. Regular physical activity is recommended and has been shown to benefit most women [73]. Healthy pregnant and postpartum women are encouraged to accumulate at least 150 min of moderate-intensity aerobic exercise per week, ideally performed over several days [18]. Beyond immediate benefits, exercise during pregnancy is associated with a reduced risk of late-onset metabolic disorders in offspring, likely mediated by epigenetic modifications during critical windows of fetal development [37,74]. Importantly, the multidisciplinary team must ensure an individualized approach, tailoring exercise prescriptions to each woman’s physiological needs, abilities, and obstetric safety profile [74].

### 4.8. Self-Monitoring of Blood Glucose

Self-monitoring of blood glucose constitutes a cornerstone of GDM management, providing essential data for the organization and titration of multidisciplinary care [75]. Accordingly, women with GDM should receive structured education to ensure consistent monitoring, as self-measured glucose values directly inform clinical decision-making and therapeutic adjustment.

According to the ADA (2026), recommended targets include fasting plasma glucose < 95 mg/dL, 1 h postprandial glucose < 140 mg/dL, or 2 h postprandial glucose < 120 mg/dL [18]. Nevertheless, glycemic thresholds are not fully uniform across international guidelines, with modest variations in postprandial targets and monitoring protocols reflecting differences in clinical interpretation and implementation strategies [41,42]. Self-monitoring is typically achieved through traditional capillary fingerstick testing or, increasingly, via Continuous Glucose Monitoring (CGM) systems. Emerging evidence suggests that CGM may provide a more accurate and comprehensive assessment of dysglycemia compared with self-monitoring of blood glucose, with improved detection of both hyperglycemic and hypoglycemic episodes. This may also enhance clinical decision-making regarding the initiation and adjustment of insulin therapy [76].

In addition, emerging research indicates that mobile health (mHealth) applications may improve glycemic control in women with GDM. mHealth-based interventions have also been associated with reduced adverse pregnancy outcomes and enhanced self-management capacity, facilitating real-time communication and data sharing between patients and HCPs [77]. Although mHealth interventions may be beneficial, it is important to consider the psychological support needs of women, as increased awareness of the risks associated with GDM may contribute to psychological distress [78].

### 4.9. Pharmacological Management

If hyperglycemia persists after 10–14 days of intensive medical nutrition therapy, pharmacological intervention is indicated [72]. This decision is guided by an endocrinologist or diabetologist in close collaboration with the obstetrician, using a “modified target level concept” that integrates fetal growth data into glycemic goals. For fetuses with asymmetrical macrosomia (abdominal circumference [AC] ≥ 75th percentile), lower glycemic thresholds are prioritized, whereas a more conservative approach is adopted for normosomic fetuses (AC < 75th percentile) to avoid overtreatment. Serial ultrasound monitoring by the obstetrician is therefore essential to calibrate these individualized targets [79].

Insulin therapy remains the gold standard for managing A2GDM, given its established safety profile in pregnancy and its inability to cross the placental barrier [72,80]. Rapid-acting analogs such as insulin lispro (Humalog) and insulin aspart (Novolog) are commonly preferred and can be administered immediately before meals, without the 10–15 min waiting period required for regular insulin [27].

In twin pregnancies, insulin requirements may be approximately double those of singleton pregnancies. Initial dosing can be estimated based on maternal weight and GA, with subsequent titration guided by glucose monitoring [38,70].

Women should be informed about the importance of consistent and correct insulin administration, its role in maintaining glycemic targets, and how it complements diet, physical activity, and lifestyle interventions [24]. This education supports safe self-administration, accurate glucose monitoring, and active engagement in overall GDM management under professional guidance [10,24].

However, some women diagnosed with A2GDM who require pharmacological treatment may not be able to use insulin due to cost, health literacy, or cultural factors. In such cases, oral agents may be considered as an alternative, although women should be informed about associated risks and the lack of robust long-term safety data in offspring. More specifically, based on high-quality data synthesized by the ADA (2026) [18], both metformin and glyburide cross the placenta and are not recommended as first-line therapy in GDM, although their clinical profiles differ substantially. Glyburide exhibits 50–70% placental transfer and is associated with less favorable neonatal outcomes than insulin, including increased neonatal macrosomia and greater abdominal circumference; however, long-term safety data for offspring are not available. In contrast, metformin readily crosses the placenta and demonstrates superior glycemic efficacy, less maternal weight gain, and lower rates of neonatal hypoglycemia compared with insulin, despite monotherapy failure rates of 14–46% and an increased risk of small-for-gestational-age infants.

### 4.10. Routine Antepartum Monitoring and Surveillance

While routine antenatal monitoring for GDM aligns with standard pregnancy protocols, HCPs must implement intensified maternal and fetal surveillance as gestation progresses. The frequency and scope of monitoring should be guided by coexisting risk factors and the severity of maternal hyperglycemia, ensuring care is appropriately escalated for women requiring pharmacological intervention [79]. In women with medically treated or poorly controlled GDM, and in the absence of additional risk factors, antepartum fetal surveillance is recommended to begin at 32 weeks of GA, with follow-up frequency individualized according to woman-specific characteristics [80].

Fetal assessment should primarily include non-stress tests (NSTs), accompanied by evaluation of amniotic fluid volume at each visit [70]. The role of fetomaternal Doppler sonography in predicting adverse perinatal outcomes in GDM pregnancies remains unclear. Doppler evaluation is generally not indicated based solely on a GDM diagnosis unless additional obstetric risk factors are present [70,81].

Additionally, routine folic acid supplementation (400–800 μg/day) and guidance on safe medication and supplement use are recommended. Comprehensive health evaluation should include overall maternal health, GDM-related comorbidities and complications, obstetric and gynecologic history, and current medications. Screening should also cover diabetes-related complications, laboratory investigations, anemia, genetic carrier status when indicated, and infectious diseases [18].

## 5. Intrapartum Care and Management

### 5.1. Labor and Delivery Management

According to the Endocrine Society, appropriate maintenance of blood glucose levels during labor ranges between 72 and 126 mg/dL. Women who required pharmacological treatment during pregnancy may need insulin administration during labor, with blood glucose monitoring every two hours [82]. HCPs attending the labor of women with GDM should be thoroughly trained to provide essential intrapartum and delivery care, ensuring maternal glycemic control and anticipating potential complications such as shoulder dystocia. They should also be able to recognize signs of hypoglycemia and other GDM-related pathological conditions in both mothers and neonates, enabling timely interventions or appropriate referrals when necessary [71,83].

A pregnant woman with GDM who is regularly monitored and properly treated can typically carry the pregnancy to term and deliver vaginally, unless additional risk factors are present. Current clinical standards from the ACOG stratify delivery timing based on the method of glycemic control. For women whose glucose levels are successfully managed with medical nutrition therapy and exercise alone (A1GDM), expectant management is generally appropriate, with delivery recommended no later than 40 (+6/7) weeks of GA. Conversely, for women requiring pharmacological intervention (A2GDM), ACOG recommends scheduled delivery between 39 (+0/7) and 39 (+6/7) weeks of GA, balancing the risk of late-gestation stillbirth with the potential for neonatal respiratory complications associated with early-term delivery [84].

However, timing of birth recommendations for women with GDM vary somewhat across national guidelines and should be interpreted in the context of local clinical protocols and healthcare resource availability [42,84].

In complex cases where glycemic control remains suboptimal despite maximal therapy, or if comorbidities such as preeclampsia or fetal growth restriction arise, delivery may be clinically indicated as early as 37 (+0/7) to 38 (+6/7) weeks of GA [84]. Elective cesarean section is recommended when the estimated fetal weight exceeds 4500 g [79,84]. When determining the timing and mode of birth, HCPs should consider glycemic control, GA, and optimal conditions for vaginal birth if chosen [73].

Women must be actively involved in decisions regarding their care plan, particularly concerning the timing and mode of delivery. Obstetricians and midwives play a critical role in supporting informed decision-making by aligning clinical recommendations with the woman’s preferences and medical needs. Endocrinologists/diabetologists, and neonatologists should also be involved in the birth plan to ensure comprehensive care for both mother and child [18,24].

### 5.2. Neonatal Management and Breastfeeding

Clinical care must balance necessary interventions with the prevention of iatrogenic harm, including avoiding unnecessary preterm deliveries, elective cesarean sections, and unwarranted separation of the mother-infant dyad. Neonates born to mothers with GDM should remain with their mothers whenever clinically appropriate, unless complications or abnormal clinical findings necessitate admission to a neonatal intensive care unit or special care setting. In addition, neonates born to women with GDM should not be discharged to community care until they are at least 24 h old and there is clinical confidence that they are maintaining stable blood glucose levels and establishing effective feeding.

Blood glucose monitoring should be routinely performed at 2–4 h after birth in infants of women with GDM. In addition, biochemical assessment should be undertaken in neonates presenting with clinical signs suggestive of polycythemia, hyperbilirubinemia, hypocalcemia, or hypomagnesemia [42]. Neonatal hypoglycemia presents variably, and otherwise healthy infants may remain asymptomatic even when plasma glucose levels are critically low during the transitional period. Clinical signs alone are not a reliable indicator of hypoglycemia [85].

Infants born to women with GDM should be fed as early as possible after birth, preferably within the first 30 min, and thereafter at regular intervals of approximately 2–3 h, until pre-feed capillary plasma glucose concentrations are maintained at a minimum of 2.0 mmol/L. Escalation of care, including tube feeding or intravenous dextrose administration, should be considered if capillary plasma glucose remains below 2.0 mmol/L on two consecutive measurements despite adequate feeding support, or in the presence of abnormal clinical signs, or when oral feeding is ineffective. In neonates exhibiting clinical signs of hypoglycemia, immediate blood glucose assessment is indicated, followed by prompt initiation of intravenous dextrose where required [42].

The benefits of breastfeeding for both mothers and infants are well-established, and it should be strongly encouraged in the context of GDM, as it may mitigate some of the short- and long-term metabolic risks associated with the condition. Evidence indicates that breastfeeding reduces the risk of childhood obesity and improves maternal metabolic outcomes [86]. However, mothers with GDM often face biological challenges, including delayed onset of lactogenesis II and higher rates of cesarean delivery, both of which are associated with lower initiation and shorter duration of breastfeeding [87]. To mitigate these risks and support neonatal metabolic stability, women may be advised to express and store colostrum antenatally from 36 weeks of GA, reducing reliance on formula or intravenous dextrose and reinforcing the breastfeeding relationship. However, antenatal colostrum expression should be considered only following individualized clinical assessment and in accordance with local protocols, with particular exclusion of women with contraindications or increased risk of preterm labor or obstetric complications [88].

Despite these recommendations, women with GDM frequently receive insufficient support from HCPs and report lower breastfeeding self-efficacy compared to women without GDM [89].

## 6. Postpartum Follow-Up and Long-Term Care

### 6.1. Postpartum Glycemic Screening

**Figure 3:**
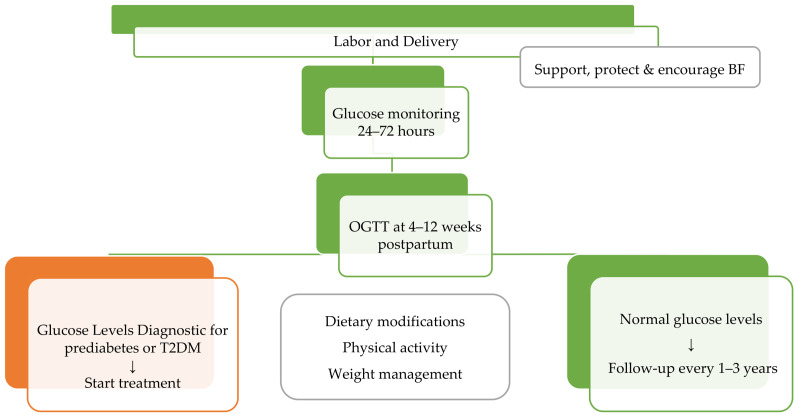
GDM postpartum follow-up and long-term care algorithm [18,38]. Abbreviation: BF (Breastfeeding); T2DM (Type 2 Diabetes Mellitus); OGTT (Oral Glucose Tolerance Test).

All women with a history of GDM should undergo glucose monitoring during the postpartum period, typically within 24 to 72 h after delivery [38]. In addition, the ADA (2026) [18] recommends that all mothers with a history of GDM undergo an OGTT between 4 and 12 weeks postpartum, followed by repeat screenings every 1–3 year intervals (Figure 3). Diagnostic criteria for T2DM are consistent with those used in the non-pregnant population and include: HbA1c ≥ 6.5%, fasting plasma glucose ≥ 126 mg/dL (after at least 8 h of fasting), 2 h plasma glucose ≥ 200 mg/dL (≥11.1 mmol/L) during a 75 g OGTT, or in individuals with classic symptoms of hyperglycemia or hyperglycemic crisis, a random plasma glucose ≥ 200 mg/dL (≥11.1 mmol/L) [18].

### 6.2. Lifestyle Counseling and Long-Term Metabolic Risk Reduction

A substantial body of evidence indicates that, in many cases, individuals with GDM do not require long-term pharmacological treatment in the postpartum period, as glucose metabolism often returns to non-pregnant physiological ranges following delivery. However, postpartum glycemic status should be formally reassessed, as a proportion of women may exhibit persistent dysglycemia or progress to T2DM. According to the ADA (2025) [18], fasting plasma glucose levels ≥126 mg/dL are diagnostic of DM, while levels of 100–125 mg/dL indicate impaired fasting glucose, consistent with prediabetes. Accordingly, medications initiated during pregnancy are generally discontinued postpartum [79,80]. However, some women continue to exhibit elevated blood glucose postpartum, and approximately 50% of women with a history of GDM develop T2DM within 5 to 10 years [68]. Therefore, postpartum care should include reminders for ongoing DM screening and provide continued support and counseling to promote healthy lifestyle behaviors after delivery. Structured follow-up programs with reminders and lifestyle guidance have been shown to improve adherence to postpartum glucose screening and support sustained behavioral change in women with GDM [90]. Women and their partners should receive education and counseling on preventive lifestyle strategies aimed at reducing the risk of developing T2DM, including guidance on nutrition, physical activity, and weight management [10,71].

## 7. Costs and Benefits

The implementation of structured, holistic, woman-centered care models GDM entails increased short-term financial demands on healthcare systems. These costs are primarily associated with antenatal education, workforce training and upskilling, development of tailored educational resources, and establishment of coordinated multidisciplinary services. Additional resource needs include staff time allocation, interpreter services, and administrative support. At the pregnant individual level, more frequent antenatal visits may impose indirect economic burdens, including transportation costs, childcare responsibilities, and income loss, which may disproportionately affect socioeconomically disadvantaged women and potentially limit engagement with care.

Despite these constraints, the benefits are substantial and multidimensional. Improved woman education and communication enhance health literacy, strengthen engagement in self-management, and increase adherence to postpartum screening, thereby facilitating early detection and prevention of T2DM. Importantly, the integration of psychosocial support addresses the emotional burden of GDM, including anxiety, stress, and diagnosis-related distress, contributing to improved coping, adherence, and maternal well-being.

From a health system perspective, improved glycemic control through coordinated multidisciplinary care and lifestyle intervention may reduce maternal and neonatal complications, with potential long-term cost savings through prevention of adverse outcomes and chronic metabolic disease. Although implementation challenges persist—particularly in settings with limited workforce capacity and training—available evidence suggests that the long-term benefits of improved outcomes and reduced complications may offset initial investments, supporting the cost-effectiveness and policy relevance of integrated, woman-centered GDM care models [24].

## 8. Accessibility and Equity in Low- and Middle-Income Settings

Substantial inequities in GDM and pregestational DM care persist across and within healthcare systems, particularly between rural and urban populations. In high-income settings, access to antenatal services is increasingly limited in rural areas, contributing to delayed diagnosis and suboptimal GDM management.

In low- and middle-income countries, implementation of multidisciplinary, holistic GDM care is constrained by structural health system limitations, including shortages of trained HCPs, limited multidisciplinary teams, inadequate referral systems, weak health information infrastructure, and restricted access to essential medications and glucose-monitoring tools. These constraints are further exacerbated by workforce instability and service disruption during economic or humanitarian crises.

From a health equity perspective, the proposed woman-centered model requires context-specific adaptation rather than direct replication. In resource-limited settings, prioritization of essential components such as midwife- and nurse-led care, task-shifting to community health workers, simplified risk stratification, structured lifestyle counseling, and low-cost capillary glucose monitoring may provide feasible alternatives to specialist-driven multidisciplinary care. Where digital health is unavailable, paper-based tracking and community follow-up may support continuity of care. Barriers to care are further reinforced by financial constraints, geographical distance, and sociocultural factors, including food insecurity, caregiving burdens, and limited access to culturally appropriate education. Importantly, similar inequities are also observed in high-income settings among migrant and refugee populations due to language barriers and reduced health literacy [24].

Overall, these considerations highlight the need for flexible, equity-oriented implementation strategies that align GDM care with available resources while preserving core principles of early detection, structured education, and continuity of care.

## 9. Limitations

This narrative review has several limitations that should be considered when interpreting its conclusions. As a non-systematic review, it was not conducted according to a pre-registered protocol and did not include a formal risk-of-bias assessment, introducing potential selection bias and the possibility of missing relevant studies. The inclusion of heterogeneous study designs, populations, and healthcare settings limits comparability and precludes quantitative synthesis, resulting in a primarily descriptive approach to synthesizing the evidence. In addition, the evidence base includes studies of variable methodological quality, including guideline-based and observational research, which may affect the robustness and consistency of the synthesis. The predominance of evidence from high-income and Anglophone settings may limit generalizability to low- and middle-income countries.

## 10. Conclusions

Emerging evidence emphasizes the necessity of a holistic, woman-centered, and multidisciplinary approach that integrates medical therapy with structured antenatal education, preventative strategies, empathetic communication, psychosocial support, lifestyle modification, and active family involvement (Table 3). Likewise, comprehensive antenatal care—encompassing individualized screening, medical nutrition therapy, glucose self-monitoring, and pharmacological intervention when required—not only ensures metabolic control but also addresses the emotional, psychological, and social dimensions of pregnancy, fostering self-efficacy, resilience, and sustainable behavioral change. Structured lifestyle counseling, ongoing glucose surveillance, and continued family-centered education reinforce healthy behaviors and support maternal well-being. Importantly, the scientific and medical communities must move beyond a narrow focus on clinical management, aligning GDM care with the recommendations of international organizations that advocate integrated, multidisciplinary, and preventive frameworks. These principles are strongly aligned with the WHO 2025 Global Guidelines on Diabetes in Pregnancy [24], which emphasize woman-centered care, multidisciplinary coordination, psychosocial support and health literacy. This convergence reinforces the relevance and applicability of the proposed framework within current international standards. Ultimately, by synthesizing preventive, educational, psychosocial, and clinical strategies within a coordinated approach, GDM management empowers women to actively engage in self-care, enhances health literacy, and optimizes both perinatal outcomes and long-term maternal and offspring health.

## 11. Implications for Practice

The effective management of GDM requires the integration of comprehensive, woman-centered strategies within routine maternity care. One important component is the incorporation of family-centered antenatal education programs that actively involve partners and close family members in the educational process. Because many recommended lifestyle modifications—such as dietary adjustments, physical activity, and glucose monitoring—take place within the home environment, engaging family members can help create a supportive setting that facilitates adherence to treatment recommendations and promotes sustainable behavioral changes throughout pregnancy.

Healthcare systems should also prioritize the development of structured multidisciplinary care pathways that ensure coordinated collaboration among obstetricians, endocrinologists, midwives, nutritionists, and neonatologists. Such approaches allow clinical management to be complemented by individualized education, nutritional counseling, and psychosocial support, addressing both the physical and emotional needs of women diagnosed with GDM. By strengthening communication between HCPs and families, these care models can improve woman engagement, enhance self-management capabilities, and contribute to better maternal and neonatal outcomes.

In addition, policies that promote holistic and woman-centered models of maternity care are essential for optimizing health outcomes. These models emphasize respectful communication, shared decision-making, and the provision of culturally sensitive educational resources tailored to different levels of health literacy. Empowering women with knowledge and practical skills can support effective self-management of GDM and reduce the risk of complications during pregnancy and childbirth. In addition, strengthening postpartum follow-up programs is another critical component of effective practice. Women with a history of GDM face a substantially increased risk of developing T2DM later in life, highlighting the importance of systematic postpartum screening, lifestyle counseling, and continued health education. Implementing reminder systems, structured follow-up pathways, and community-based support services may improve adherence to postpartum glucose testing and encourage the maintenance of healthy lifestyle behaviors after delivery.

Finally, continued research is essential to further strengthen evidence-based GDM care. Future studies should focus on evaluating the effectiveness of family-centered educational interventions, multidisciplinary care models, and digital health tools in improving glycemic control, psychological well-being, and long-term metabolic outcomes. Research exploring culturally adapted interventions and strategies to improve postpartum follow-up adherence is also needed. Such evidence will support the development of more effective and accessible care models that address both the clinical and psychosocial dimensions of GDM management.

## Figures and Tables

**Table 1:** Desired weight gain according to Institute of Medicine/National Research Council guidelines [32].

Classification	Pre-Pregnancy BMI	Recommended Weight Gain
Underweight	<18.5 kg/m^2^	12.5–18 kg
Normal weight	18.5–24.9 kg/m^2^	11.5–16 kg
Overweight	25.0–29.9 kg/m^2^	7–11.5 kg
Obese	≥30.0 kg/m^2^	5–9 kg

**Table 2:** World Health Organization recommendations for GDM Antenatal Education [24].

Aspects of Education
Preconception weight management and appropriate gestational weight gain; Managing glycemia; Healthy lifestyle changes (diet and physical exercise); GDM aspects (the effects of DM in pregnancy on maternal, fetal, newborn and child health outcomes); The need for additional monitoring of fetal growth and well-being.

Abbreviations: DM (Diabetes Mellitus); GDM (Gestational Diabetes Mellitus).

**Table 3:** Summary of holistic management approaches for GDM.

Care Domain	Key Interventions/Strategies	Target Outcomes
Risk Profiling and Preconception Care	Early assessment of modifiable (BMI, lifestyle) and non-modifiable risk factors; preconception glycemic screening; counseling on nutrition, physical activity, and weight management	Early identification of high-risk pregnancies; prevention of GDM onset; reduction in adverse maternal/fetal outcomes
Antenatal Education	Structured, individualized programs; culturally tailored information; family involvement in sessions; guidance on blood glucose monitoring, diet, and physical activity	Improved self-management; enhanced knowledge and adherence; reduced maternal stress; support from home environment
Psychological Support	Routine assessment of mental well-being; counseling; cognitive-behavioral interventions; peer support networks	Reduced anxiety, stress, and depression; improved adherence to lifestyle and pharmacologic therapy
Medical and Nutritional Management	Lifestyle modifications (diet, physical activity); medical nutrition therapy; pharmacological intervention (insulin if needed); daily glucose self-monitoring; multidisciplinary team approach	Glycemic control; prevention of macrosomia and neonatal complications; optimal maternal weight gain
Intrapartum Care	Blood glucose monitoring during labor; coordination among obstetricians, midwives, endocrinologists, neonatologists; planning for delivery mode based on glycemic control and fetal growth	Safe delivery; minimized neonatal hypoglycemia; reduced obstetric complications
Neonatal Management and Breastfeeding	Early skin-to-skin contact; monitoring neonatal glucose; antenatal colostrum expression; support for breastfeeding	Stable neonatal glucose; improved breastfeeding initiation and duration; long-term metabolic protection
Postpartum Follow-Up	OGTT 4–12 weeks postpartum; repeat screening every 1–3 years; life style counseling; family education; reminder systems	Early detection of T2DM; sustained healthy behaviors; reduced long-term metabolic risk

Abbreviations: BMI (Body Mass Index); GDM (Gestational Diabetes Mellitus); OGTT (Oral Glucose Tolerance Test); T2DM (Type 2 Diabetes Mellitus).

## Data Availability

No new data were created or analyzed in this study.

## Glossary

A1GDM	DM that is first diagnosed during pregnancy and is managed entirely through lifestyle changes (diet and exercise) without the need for medications like insulin or oral glucose-lowering agents (Class A1 GDM) [84].
A2GDM	DM that is first diagnosed during pregnancy requiring pharmacological treatment, including insulin and/or oral glucose-lowering agents, in addition to lifestyle modification (Class A2 GDM) [84].
High-risk population	Pregnant individuals with an increased risk of developing insulin resistance and/or hyperglycemia [43].
Overt DIP	A DM first recognized during pregnancy that is clearly pre-existing. A pregnant individual meets any of the following criteria: FPG ≥ 7.0 mmol/L or 2hPG ≥ 11.1 mmol/L following a 75 g two-hour OGTT; HbA1c ≥ 6.5% (≥ 48 mmol/mol); and/or random plasma glucose ≥ 11.1 mmol/L in the presence of clinical signs or symptoms indicative of hyperglycemia [43].
Pre-existing DM	DM (T1DM or T2DM) diagnosed prior to pregnancy [43].

## Abbreviations

The following abbreviations are used in this manuscript:

AC	Abdominal circumference
ACOG	American College of Obstetricians and Gynecologists
ADA	American Diabetes Association
ADIPS	Australasian Diabetes in Pregnancy Society
BF	Breastfeeding
BMI	Body Mass Index
CGM	Continuous Glucose Monitoring
DIP	Diabetes in Pregnancy
DM	Diabetes Mellitus
FPG	Fasting Plasma Glucose
GA	Gestational Age
GDM	Gestational Diabetes Mellitus
HbA1c	Hemoglobin A1c
HCPs	Health Care Professionals
HMGB1	High Mobility Group Box 1
IADPSG	International Association of Diabetes and Pregnancy Study Groups
IDF	International Diabetes Federation
NICE	National Institute for Health and Care Excellence
NSTs	Non-stress tests
OGTT	Oral Glucose Tolerance Test
PAPP-A	Pregnancy-Associated Plasma Protein A
PCOS	Polycystic Ovary Syndrome
T1DM	Type 1 Diabetes Mellitus
T2DM	Type 2 Diabetes Mellitus
WHO	World Health Organization
